# Interplay of Inflammatory, Antigen and Tissue-Derived Signals in the Development of Resident CD8 Memory T Cells

**DOI:** 10.3389/fimmu.2021.636240

**Published:** 2021-06-21

**Authors:** Curtis J. Pritzl, Mark A. Daniels, Emma Teixeiro

**Affiliations:** Department of Molecular Microbiology and Immunology, School of Medicine, University of Missouri, Columbia, MO, United States

**Keywords:** resident memory, inflammation, antigenic stimulation, tissue-derived signals, memory differentiation

## Abstract

CD8 positive, tissue resident memory T cells (T_RM_) are a specialized subset of CD8 memory T cells that surveil tissues and provide critical first-line protection against tumors and pathogen re-infection. Recently, much effort has been dedicated to understanding the function, phenotype and development of T_RM_. A myriad of signals is involved in the development and maintenance of resident memory T cells in tissue. Much of the initial research focused on the roles tissue-derived signals play in the development of T_RM_, including TGFß and IL-33 which are critical for the upregulation of CD69 and CD103. However, more recent data suggest further roles for antigenic and pro-inflammatory cytokines. This review will focus on the interplay of pro-inflammatory, tissue and antigenic signals in the establishment of resident memory T cells.

## Introduction

Over the course of an infection, naïve CD8 T cells become activated in the lymphoid tissues and differentiate into CD8 effector T cells. As effector T cells abandon the secondary lymphoid organs and migrate to tissue, they need to integrate a multitude of signals coming from cytokines, chemokines and antigen in order to gain access to infected cells, clear the pathogen and differentiate into memory T cells. Among the T cell responders with effector function, the vast majority die and only a few persist as memory T cells. We do not yet fully understand what endows T cells with the potential to become memory T cells, although we do know that the level of exposure to antigenic and pro-inflammatory signals play an important role (1–6). We also know that a balance in the level of a set of transcription factors is crucial (i.e. Eomes/T-bet, Bcl-6/Blimp-1, Id-2/Id-3, ZEB1/ZEB2, BACH/AP-1, NR4A1/IRF4) (7, 8); that specific costimulatory and homeostatic cytokines signals impart maturing memory cells with longevity properties (9, 10); and that dramatic metabolic and epigenetic changes are essential (11, 12). Precursors of memory T cells (or MPECs) have been well defined as KLRG1^lo^ and IL-7R^hi^ (2) and are readily present early in the immune response albeit at small frequencies. Yet, as most of antigen specific-T cell responders progress through the immune response and die off (Short lived effectors/SLECS KLRG1^hi^ IL-7R^lo^ expressors), MPECs continue their process of maturation towards memory. Consequently, T cell memory is the result of a combination of early signals which configure the transcriptional/epigenetic memory program, and late signals that during the same immune response help to fully execute this program (13, 14). T cell memory differentiation becomes even more complex when considering that memory T cells come in different “flavors” (T cell memory subsets) and with different benefits (T cell memory functions and locations). Thus, a T cell transitioning to memory, may become a central memory (T_CM_), an effector memory, (T_EM_), a stem-cell memory (T_SCM_), or a resident memory (T_RM_). Each population has evolved to fill a specific niche required to protect the host. T_CM_ (CCR7^+^ CD62L^+^ expressors) circulate between the blood and secondary lymphoid tissues and retain an extraordinary proliferative potential. T_EM_ (CCR7^-^CD62L^-^), in turn, circulate between the blood and peripheral tissues and are very efficient at exerting immediate effector functions upon antigen restimulation [reviewed recently in (15)]. T_SCM_ have been described in humans (CD122^+^, CD95^+^, CCR7^+^, CD62L^+^, CD45RA^+^, CXCR3^+^) and share the proliferative, self-renewal and pluripotency potential of T_CM_ cells (16).

**Figure 1:**
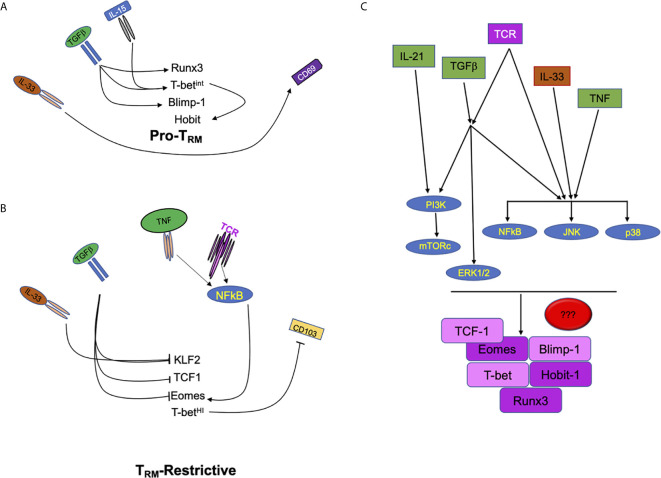
Extracellular factors regulate multiple signals in CD8 T cells to drive or repress T_RM_ development. **(A)** Schematic of signals including IL-33, TGFβ, and IL-15 which promote the development of tissue resident T cell memory through the increase of transcription factors Runx3, Hobit, Blimp1, and the tuning of T-bet expression. **(B)** Tissue cytokines such as IL-33 and TGFβ also inhibit transcription factors (KLF2, TCF1, and Eomes) that can restrict the development of CD8 T_RM_. In contrast, pro-inflammatory cytokine and antigenic/T cell receptor signals can modulate the expression of Eomes which can, then, interfere with CD8 T_RM_ development. **(C)** Signaling crosstalk between pro-inflammatory, tissue and antigenic signals. PI3K, MAPKs (ERK, JNK and p38 MAPK) and NFκB are potential nodes where extracellular cues converge to tune CD8 T_RM _programming, differentiation and maintenance.

Tissue resident memory T cells persist in the peripheral tissues following infection and act as front-line sentries against pathogen re-infection. The response of CD8 T_RM_ triggers fast innate (17–19) and adaptive immune responses in the site of re-infection (20). Furthermore, CD8 T_RM_ have also been linked to defense against tumors, with its presence correlating with good prognosis (19, 21, 22). CD8 T_RM_ are present in almost every tissue, including secondary lymphoid organs (23). However, there is also phenotypic diversity of the T_RM_ subset depending on the tissue. This suggests that local tissue signals may play a critical role in positioning T_RM_ in specific locations to perform specialized functions (24). In spite of how much we have learned in recent years about T_RM_, there is still little known about how cytokines, antigens and other tissue signals “crosstalk” intracellularly to program the generation and maintenance of CD8 T_RM_ (**Figure 1**). In this review article we will discuss how much the field has advanced in this aspect and point out to the gaps that still remain uncovered.

## Tissue Resident Memory CD8 T Cells

As mentioned before, tissue resident memory CD8 T cells have been found in peripheral healthy tissues such as lung, brain, gut, liver, skin, oral, nasal and female reproductive tract mucosal tissue, and also in tumors, transplants and organs subjected to autoimmune reactions (23). Most interestingly, tissue resident memory T cells also re-populate tissue draining lymph nodes upon antigen recall. Even at the memory stage, tissue T_RM_ can occupy local draining lymph nodes, most likely, to warrant extended protection (25, 26). All together this puts T_RM_ as the most abundant memory T cell in our bodies and especially so as we age. In mice, it is difficult to evaluate the lifespan of T_RM_ beyond one year. However, in humans, it has been shown that T_RM_ are stably maintained from childhood well into old age, at levels that are tissue specific (27, 28). Surprisingly and in contrast to mice (where naïve T cells largely reside in lymphoid organs), in humans naïve T cells are also long-term resident of tissues, although they are quickly outnumbered by memory T cells in mucosal sites (29). Resident memory T cells are extremely efficient at mounting protective innate and adaptive secondary responses upon re-infection (17, 30) and can control pathogen spread without the need of other T cell memory subsets (31). Yet whether this helps to spare the naïve and central memory population in lymph nodes from activation, and further maintain diversity in the T cell repertoire remains to be shown.

T_RM_ ontogeny is also still poorly understood as well as the relationship of the T_RM_ subset with the other T memory subsets. Initially MacKay, Carbone and Gebhardt described KLRG1^lo^ epithelium expressors that encounter IL-15 and TGFβ signals as precursors of skin T_RM._ This led to the idea that T_RM_ cells deviate from the T effector differentiation path once in tissue (32, 33). More recently, other studies have confirmed that even before tissue entrance circulating T cells can commit to the T_RM_ fate. This is readily concluded when considered that: (1) T_CM_ and T_RM_ share a common clonal origin (34); (2) even at the naïve stage, T cells can be pre-condition to “walk” the T_RM_ differentiation journey (35) and (3) that circulating effectors with a skewed T_RM_ transcriptional profile that preferably become T_RM_ exist (36). Whether this also applies to the ontogeny of T_RM_ in other tissues is still uncertain. Indeed, in contrast to the skin T_RM_ studies, scRNA sequencing studies in the gut have identified T_RM_ precursors in tissue very early upon infection (37). From all these data, one thing is still clear, regardless of the potential for becoming T_RM_, circulating effectors will not be able to fulfill this potential unless exposed to tissue signals.

At the point T cells commit to the T_RM_ fate, are they deadlock in this identity? or on the contrary, do they retain pluripotency to generate other T cell memory subsets upon recall? Fonseca et al. answered this question recently and provided evidence supporting the idea that T_RM_ cells are not completely locked into the resident lineage. Upon rechallenge, ex-T_RM_ cells epigenetically retained the potential to become T_CM_ and T_EM_ (38), however, they preferentially re-differentiate into T_EM_ and T_RM_ that homed back to their original tissue (38, 39).

Another important issue in the field is T_RM_ diversity of heterogeneity. T_RM_ diversity is defined by changes in transcription profile, phenotype, location and function (37). However, despite the heterogeneity within the T_RM_ compartment, all T_RM_ share a specific transcriptional profile characterized by expression of Runx3, Blimp-1, and Hobit and reduction of Eomes, T-bet, and KLF-2 levels (40–43) (**Figure 1**). This transcriptional profile enables the expression of molecules that permit recruitment and lodging to tissue in addition to special adaptation to unique tissue signals for T_RM_ survival. What is less known is how the different signals a T cell encounters in its journey to T_RM_ regulate this transcriptional program.

A more precise view of T_RM_ development is arising. Cumulative evidence supports a multistep differentiation process where T cells have the potential to enter in the T_RM_ path at different stages (naïve, in circulation, in tissue). Yet how much the quality or amount of signals a T_RM_ precursor receives conditions its resident potential is unclear. Additionally, it is still ill-defined whether the same signals regulate T_RM_ development, maintenance, function, retrograde migration to draining lymph nodes and/or pluripotency upon recall. Initial findings pointed to various cytokine signals and antigen within local tissues as main triggers to support CD8 effectors to CD8 T_RM_ differentiation. TGFβ has been shown to be a major contributor to this pathway along with IL-33 and IL-15. Roles for both antigenic stimulations along with inflammatory signals such as IL-12, IL-21, and TNF have been linked to the regulation of CD8 T_RM_ development as well (**Figure 1**).

## Tissue Signals Involved in Cd8 T_rm_ Development

Tissue cytokines have been shown to act synergistically in establishing the resident memory phenotype in tissues such as the gut, skin, brain, and the lungs (40, 44–49). Hereafter, we will discuss what it is known of how each one of these signals contribute to T_RM_ development and maintenance and discuss the synergism of the signaling pathways they trigger.

### TGFβ Signaling

TGFβ is a crucial cytokine for T cell development and differentiation. TGFβ is involved in thymic development, in the maintenance of naïve T cells, and also in CD8 T cell effector activation (50, 51). Seemingly, TGFβ has also been linked to the formation of CD8 T_RM_ in different organs such as skin, the gut and lung (32, 44, 45, 52, 53).

Although TGFβ and its receptor are ubiquitous in many cells, TGFβ activity is tightly controlled at multiple levels. At the extracellular level, TGFβ activity depends on induced cleavage of latent TGFβ that is associated to the extracellular matrix or presentation by cells (such as T regs, epithelial cells, fibroblasts, keratinocytes or DCs). Large latent TGFβ can be cleaved by ECM proteases. Alternatively, it can bind to integrin receptors in the membrane of cells, which *via* the actin cytoskeleton promote a conformational change in TGFβ that enables the mature TGFβ release process (54). TGFβ modulates T_RM_ in a manner that is contingent on the presence of immune cells expressing a specific set of integrin receptors. Thus, in the draining lymph nodes of the skin, specialized migratory DCs that express α_v_ integrins present active TGFβ to naïve T cells and pre-condition them to become epithelial CD8 T_RM_ (35). More recently, Hirai et al. provided data showing that keratinocytes activation and presentation of TGFβ to fully matured skin CD8 T_RM_ is crucial for their maintenance. Especially, if these T_RM_ had been generated in a bystander manner. Even more striking is that skin CD8 T_RM_ produce their own TGFβ, thereby, contributing to their own maintenance (55). These new compelling roles of TGFβ in skin CD8 T_RM_ add to the already known role of TGFβ in CD8 T_RM_ differentiation (32, 40). However, they also open up new exciting questions. For instance, do these new roles of TGFβ apply to T_RM_ in other tissues? Or what is the relative contribution of autocrine CD8 T_RM_ TGFβ to T_RM_ lineage identity versus T_RM_ survival?

CD103 is one of the most thoroughly described targets of TGFβ in T_RM_ cells (32, 44, 45, 52, 56, 57). CD103 is an integrin (alpha E) that associates with integrin beta 7. The αEβ7 integrin complex binds to E cadherin and facilitates migration and retention of CD8 T cells (32, 58, 59). While not exclusively required for development of all T_RM_ cells, CD103 has an important role in the establishment of tissue residency within certain tissues, such as gut and skin. Sheridan et al., showed that upon oral *Listeria monocytogenes* infection, the majority of the intestinal effector cells rapidly upregulated CD103, but this population was lost when TGFβ signals were blocked (52). In the lung, it has been reported that CD1c+DCs control CD103 expression on CD8 T cells, enabling their accumulation in lung epithelia through a membrane-bound TGFβ dependent process (60). Lack of access to active TGFβ from fully matured skin CD8 T_RM_ also lead to a loss of CD103 expression, although this loss appears to correlate better with the amount of active TGFβ than with a defect in CD8 T_RM_ differentiation (55). This raises the question as to whether CD103 only provides signals for localization or whether it also activates signal transduction pathways that promote T_RM_ lineage stability. The former is supported by the fact that in several tissues (female reproductive tract, liver, lung, and lamina propria) CD103 is not expressed by all resident memory cells (23, 61). It is also important to mention that CD103 is an integrin able to trigger bidirectional signaling and that it can cooperate with TCR signals to enable T cell migration and effector function (62). This suggests that synergism between antigenic and integrin signaling at the epithelium may be relevant for T_RM_ maturation.

Despite the important role of CD103 in CD8 T_RM_ adhesion, migration and retention in TGFβ rich environments, TGFβ receptor deficient cells are more compromised than CD103 deficient T cells for tissue long-term retention (44). Thus, the TGFβ role in CD8 T_RM_ development must be broader than CD103 regulation. Indeed, several studies have pointed to other roles. TGFβ has been found to induce apoptosis of short-lived effector cells (SLECs) by antagonizing the survival effects of IL-15 (63). Since CD8 T_RM_ maintenance in some tissues depends on both cytokines, it is possible that TGFβ contributes to the removal of SLECS, thereby favoring MPEC survival and retention in tissue (**Figure 1**). Comparative *in vitro* analysis also demonstrates a great overlapping between T_RM_ and TGFβ transcriptional signatures (64). More precisely, TGFβ signaling regulates the expression of transcription factors involved in T_RM_ development, such as Runx3 (65) and Blimp1 (66) and repress transcription factors (Eomes, TCF1, and T-bet) (40, 46), which are classically associated with CD8 terminal effector and central memory differentiation (5, 67–70). Achieving the right balance in the levels of all of these transcription factors appears to be crucial for the development of CD8 T_RM_. Thus, while some T-bet expression is necessary for the expression of IL-15Rβ to receive sufficient IL-15 signals to lodge and survive in tissue (40, 47), over activation of T-bet can also result in the loss of CD103 expression (40, 71). Similarly, high levels of Eomes have been shown to repress T_RM_ development (40). It is still unclear how these transcriptions factors cooperate to establish the T_RM_ program. Yet, they seem to operate under different transcriptional rules than those regulating effector CTL differentiation (where all transcription factors work together in a synergistic way) (68).

Another role of TGFβ is to control tissue lodging by suppressing the expression of Krupple-Like Factor 2 (KLF2), which in turn regulates the expression of S1PR1 (42). Skon et al. reported that TGFβ can control the lodging of CD8 T_RM_ by downregulating KLF2 in a PI3K/Akt dependent manner (42). Curiously, canonical TGFβ signaling classically occurs through the induction of the SMAD pathway and involves formation of activated Smad2/3/4 complexes (54). However, Smad4 appears to be dispensable for CD8 T_RM_ development (72, 73). This implies that non canonical TGFβ signaling may be more important than anticipated for CD8 T_RM_. TGFβR engagement can activate MAPKs p38, JNK, and ERK, NFκB, PI3K, and mTOR signaling pathways independently of Smad proteins (72–74), although the role of these pathways in CD8 T_RM_ remains elusive. MAPKs (**Figure 1**), in particular, might be especially relevant as recent transcriptional studies have found an association between JunB and FosL and T_RM_ differentiation (37).

Lastly, it is important not to underestimate the crosstalk of TGFβ with other tissue signals which may further tune TGFβ signaling and pay attention of how these signals interaction may account for further diversity or differences in CD8 T_RM_ longevity and/or function (54, 74).

### IL-33 Signaling

Along with TGFβ, IL-33 has also been involved in the establishment of CD8 resident memory. IL-33 is a part of the IL-1 family of cytokines. It is expressed by non-hematopoietic cells, constitutively in epithelial cells and inducible in activated DCs, necrotic cells, and tumor cells. It works as an alarmin in response to infection or injury [reviewed in (75, 76)]. CD8 T cells express low levels of the IL-13R or ST2 but IL-33 signaling is still important for effector function (77) and antiviral protective responses (78). Following the initial characterization of CD8 T_RM_, Casey et al. showed in *in vitro* experiments, that IL-33 could act synergistically with TGFβ to induce CD69 among CD8 T cells in the gut (45). The role of IL-33 was further defined to include the down regulation of KLF2, again in synergism with TGFβ (42). More recently, Harty’s group explored the role of IL-33 in the formation and maintenance of lung CD8 T_RM_ *in vivo*. They found that when ST2 was blocked with a neutralizing antibody, the accumulation of influenza specific CD8 T_RM_ was significantly reduced. Yet no effect on conversion to a T_RM_ phenotype was observed (79). In another study, McLaren et al. also showed a loss of CD8 and CD4 T_RM_ (CD69^+^CD103^-^ or CD69^+^CD103^+^) in the lungs and salivary glands of IL-33-deficient mice upon MCMV infection (49). Collectively, these data strongly support a critical role of IL-33 in the establishment of the T_RM_ pool in the lung, although whether this role impinges on CD8 T_RM_ differentiation, maintenance and/or recruitment is unclear. Similarly, it is still unknown whether IL-33 impacts CD8 T_RM_ in a CD8 T cell intrinsic manner or through an indirect mechanism. The *in vitro* experiments mentioned above (45), however, point out to a direct role in synergism with TGFβ.

IL-33 signals through MyD88/NFκB can inhibit TGFβ signals through Smad6/7 (74). Furthermore, IL-33 can synergize with IL-12 to promote the expression of T-bet and Blimp-1 while repressing Eomes and TCF-1 (77) (all transcription factors linked to CD8 T_RM_ differentiation) (**Figure 1**). Taking all together (**Figure 1**), it is tempting to speculate that CD8 T_RM_ differentiation and maintenance will be likely dependent on the relative levels of these cytokines in tissue and how their signaling networks crosstalk.

## Inflammatory Signals and Resident Memory

### Tumor Necrosis Factor

TNF is a cytokine that has pro- and anti- inflammatory functions. TNF is first expressed as a biological active transmembrane homotrimer, which can either be released after cleavage and bind to TNFR1 or TNFR2 or remain bound to the membrane and signal upon binding to TNFR2. TNFR1 is expressed universally on almost all cell types, whereas TNFR2 is mainly restricted to immune cells and some tumor cells. TNF, by contrast, can be produced by T and B cells and innate immune cells (dendritic cells, monocytes, neutrophils, mast cells). TNF is an inflammatory mediator that is heavily induced upon infections such as influenza or tuberculosis but their long -term effects are frequently associated with pulmonary diseases such as asthma, COP, ALI, and ARDS (80). In T cells, TNF can promote the activation and proliferation of naïve and effector T cells, but it also promotes cell death of highly activated effector T cells, further determining the size of the memory T cell pool (81). *In vitro* studies have shown that TNF can synergize with TGFβ and IL-33 to regulate the expression of molecules associated with a T_RM_ signature (CD103, CD69 and Ly6C) in the gut, as well as regulate the expression of the transcription factor KLF-2 (facilitating the retention of T_RM_ in tissue) (42, 45, 82). Additionally, in experiments aiming to test the role for cytokines in the conversion of circulating memory T cells to lung T_RM_, the authors found that neutralizing TNF levels resulted in a significant reduction in the frequency of CD8 T_RM_ in the parenchyma (79). Altogether, these studies strongly support a role for TNFα in the establishment of T_RM_, however, whether TNF effects act directly on CD8 T_RM_ precursors *via* their TNFR1 or TNFR2 or indirectly *via* other cells it is still unclear. A study showed that mice lacking TNFR1 expression were inefficient at controlling vaccinia virus in the skin, rather due to defects in resident innate cells and not to the generation of skin memory T cells (82). On the other side, other studies have implicated both TNFR1 and TNFR2 in survival of airway CD8 effectors during influenza infection (83) and also in the generation of memory T cells (81, 84). Thus, when considering the multifaceted roles of TNF signals in the progressive differentiation of CD8 T cells, more studies are needed to assess when and how TNF impacts CD8 T_RM_ and if this happens for all tissues.

Members of the TNF superfamily OX-40 (85), 4-1BB (86, 87) and LIGHT (88) have also been linked to the establishment of CD8 T_RM_. 4-1BB and LIGHT appear to be crucial for the survival of effector CD8 T cells as they differentiate to T_RM_ (86–88), whereas OX40 signals rather seem to impact the generation of effector and, therefore, accumulation of memory T cells in tissue. One feature in common among all members of the TNF superfamily (TNF included) is the activation of NFκB PI3K, Akt, MAPK and JNK pathways (89), which most likely allow for enhanced survival. However, all TNF superfamily members are also notorious for their dependence on TCR (for costimulatory functions or expression) or cytokine signals (i.e. TNF synergism with TGFβ signals). This points to a more complex picture regarding how all these factors play together in tissue as T cells differentiate and are maintained as CD8 T_RM_ (**Figure 1**). Given the therapeutic value of neutralizing antibodies and fusion proteins targeting TNF family members to decrease inflammation, addressing these gaps of knowledge will aid to improve current strategies directed to boost CD8 T cell immunity in organs or tumors. Similarly, and because anti-TNF treatments are often administered to diminish inflammation in diseases such as Crohn’s and rheumatoid arthritis (90–92), knowing the impact of these treatments in the generation and maintenance of the T_RM_ pool in patients is also important.

### Interleukin 12, Type I IFN, IL-18, IL-21, and IL-6

Both IL-12 and Type I IFN are the prototypic pro-inflammatory cytokines that provide signal 3, which with signal 2 (costimulation) and signal 1 (antigen/TCR) enable full effector and memory differentiation (93–96). It has also been shown that high levels of these pro-inflammatory cytokines skew effector T cells away from memory (2, 97, 98). Intestinal proinflammatory microenvironments have elevated IFN-β and IL-12 and several studies have shown that both cytokines are critical drivers of CD8 T_RM_ in the gut. Bergsbaken et al. identified intestinal CCR2^+^ macrophages as the main source of both pro-inflammatory cytokines in the gut and showed that either deletion of these innate population or deletion of the receptors for IL-12 or Type I IFN on CD8 T cells could severely reduce the differentiation and persistence of gut CD103^-^CD69^+^ CD8 T_RM_ cells. Importantly, this was not a consequence of defects in expansion or survival of effector CD8 T cells early in the infection, but rather it was connected to the integration of pro-inflammatory cytokine signals (IL-12, IFNβ, or IL-18) and TGFβ signals in tissue (99). Another report has also shown that IL-12 acting together with IL-15 and CD24 signals is essential for the development of potent CD8 resident memory responses in the skin. In this case, a migratory BATF3^+^ dendritic cell population was the main source of IL-12. When tissue IL-12 signaling was inhibited using antibody blockade, sub-optimal CD8 T_RM_ generation was observed in the skin of vaccinia virus-infected mice (100).

IL-12 can also contribute to the establishment of skin CD8 T_RM_ through the expression of the adhesion receptor CD49a, which is specifically critical for CD8 T_RM_ persistence and IFNγ production upon recall (101). At the transcriptional level, IL-12 is a known regulator of master regulators of CD8 T_RM_ Eomes, T-bet and Blimp-1 (102, 103). T-bet is required for the expression of CD122 and input of IL-15 signals necessary for CD8 T_RM_ survival (40, 47), suggesting that IL-12 indirectly facilitates CD8 T_RM_ survival. At the same time, high levels of T-bet may be detrimental for CD8 T_RM_ (40). Since all the studies so far have evaluated the blockade of IL-12 signals to test the role of this cytokine in CD8 T_RM_, it would be interesting to test whether high levels of IL-12 (which can naturally occur in cytokine storms) could be detrimental, perhaps by exceeding the T-bet threshold that transcriptionally supports T_RM_ (40, 104).

IL-21 is another pro-inflammatory cytokine that is primarily expressed by CD4 T cells, although macrophages, NKT, B, DC, and CD8 T cells can express it at low levels (105). Recently, it has been shown that IL-21R CD8 T cell intrinsic signaling is important for the development of lung and brain CD8 T_RM_ *via* oxidative metabolism (106, 107). IL-21 has been shown to synergize with other cytokines (IL-2, IL-15, IL-10) and TCR signals for regulating CD8 T cell differentiation (108). IL-21R, in turn, transduces signals *via* STAT-1/3/5, but it also shares the activation of PI3K and MAPK with other tissue signals (antigen, TGFβ, TNF), establishing in this way a potential system of check and balances that warranties CD8 T_RM_ [reviewed in (105)] (**Figure 1**).

IL-6 shares functional features with IL-21, and it is produced in certain tissues (bone, lung, liver, adipose tissue, muscle) to fulfill homeostatic functions as well as in response to infection, cancer and tissue injury (109–111). IL-6 signals through STAT3 and together with TGFβ is primordial for Th17 differentiation (112). Furthermore, IL-6 stimulates the production of IL-21 by CD4 T cells (113) and exerts a pro-survival role that can impact the effector/memory population in the context of infection (114, 115). In CD8 T cells, IL-6, together with IL-15 and IL-7, contributes to CD8 T cell proliferation and effector function (116) and to the generation of super IL-21 producer CD8 T cells that can then, help B cells in the lung (117). The connection between IL-6 and tissue resident T cell memory is still poorly understood, although a recent report has identified a distinct population of memory helper CD8 T cells in humans that singularly express IL-6R and exhibit a skin T_RM_ transcriptional signature (118). Interestingly, these IL-6R CD8 memory T cell population is altered in psoriasis (118) and asthma (119), although a role for these type of T cells during infection is still lacking.

Experimental evidence supports that an interaction between local tissue signals and pro-inflammatory cytokines is essential for the establishment of CD8 T_RM_ during infection. Yet, often in systemic infections, cancer therapies (CART) and autoimmunity (rheumatoid arthritis, psoriasis), levels of these pro-inflammatory cytokines or signaling can become dysregulated and cause disease. IL-6 is, indeed, together with TNF, IL-1, IL-18, IL-33, IFNγ a soluble mediator of cytokine storms (120) in mucosal tissues, although whether high levels of inflammatory cytokines are beneficial for CD8 T_RM_ establishment or maintenance still remains to be investigated.

## Homeostatic Signals IL-7, IL-15 and IL-10

Dendritic cells are key to initiating immune responses and often for directing those responses to the appropriate tissues *via* delivery of antigen, co-stimulation and pro-inflammatory cytokines. What is less studied is how their contribution to homeostatic signals shape the immune response. Iborra et al. recently showed that DNGR-1+ dendritic cells cross present antigen and produce IL-12, IL-15 and CD24 signals which were required for CD8 T_RM_ formation in the skin and lungs (100). IL-15, together with IL-7, is a homeostatic cytokine whose role in T_CM_ and T_EM_ cell memory maintenance is well established (121–123).

In the context of resident memory, IL-7 is almost dispensable while IL-15 has been shown fundamental for survival of CD8 T_RM_ in some tissues (such as skin, kidney, lung and salivary glands but not in FRT, gut, pancreas) (32, 47, 124). In the skin, IL-15 contributes to lodging and maintenance of CD8 T_RM_ by keeping balanced levels of T-bet and the transcription factor Hobit (40, 43). Hobit, in turn, is expressed exclusively in the resident memory population and has the potential to bind to regulatory regions of TCF1, KLF2 and S1PR1, all crucial for CD8 T cell tissue migration (43). In the liver, skin, and small intestine, Hobit has been shown to act in conjunction with Blimp-1 to drive T_RM_ development as well (43). However, in the lung, Blimp-1, rather than Hobit drives T_RM_ formation (125). This is despite the fact that persistence of a subset of lung CD8 T_RM_ (CD103^+^CD69^+^) is completely dependent on IL-15 (40). Interestingly, the patterns of Hobit expression and function in mice and humans are different (126), but whether the results in the mouse models remain true in humans will require further investigation. Contrary to Hobit, Blimp-1 promotes CD8 T_RM_ development in the lung while reducing the generation of CD8 T_CM_. This is particularly critical for CD103^+^ CD25^+^, but not CD103^–^ CD25^-^ lung T_RM_ (125). While this points out to a potential role of IL-2 and IL-15 in regulating the levels of Blimp-1 the evidence remains controversial. *In vitro* studies have attributed a role for IL-2, but not IL-15, in the induction of Blimp-1 (127). By contrast, *in vivo* studies delivering IL-15 complexes have clearly shown that acute exposure (but not prolonged) to IL-15 signals can promote Blimp-1 expression (128). As IL-12 is also an inducer of Blimp-1 (103), it is possible that specialized DCs able to produce IL-15 and IL-12 (100), together with IL-2, contribute to the induction of Blimp-1 and generation of lung CD8 T_RM_ in sites with residual inflammation.

Another cytokine that is often induced in response to infection is IL-10. CD4 regulatory T cells (Tregs) are producers of IL-10 (129). Both, Tregs and IL-10, play a critical role late in the immune response in the generation of memory CD8 T cells (130). Similarly, Type 1 Tregs (T-bet-) also promote the generation of CD8 T_RM_. In this case a distinct role for IL-10 was not clearly identified. Instead, the authors found that CD4 Tregs express CXCR3 and by positioning themselves close to CD8 T cells make functional TGFβ available to promote their T_RM_ differentiation (131).These findings were consistent with previous studies indicating that TGFβ-dependent production of TGFβ resulted in increased expression of CD103 on brain CD8 T cells upon CNS infection (132).

## T Cell Receptor Signals and Resident Memory CD8 T Cells

T cells recognize pathogenic or self-antigens *via* their T Cell Receptors (TCRs). TCR signaling is critical for memory T cells (5). Strikingly though, while T cell proliferation and some effector functions are supported by strong antigenic signals, T cell memory ensues regardless, in response to both strong and weak antigens (1, 6). These studies mainly looked at central and effector memory differentiation and found that weak TCR signals specifically favor central memory development *via* expression of high levels of Eomes. Moreover, TCR signal strength inversely regulated the input of inflammation by controlling the expression of inflammatory cytokine receptors and enabling a higher frequency of CD8 T cells that have been stimulated by weak antigens to become central memory T cells (1, 133). In the case of resident memory differentiation, the role of TCR signaling has been largely overlooked until recently. Fiege et al. have shown that while both high and low affinity TCR stimulation support the formation of CD8 T_RM_, low affinity TCR signals favored the resident memory population (134) mirroring what happens for central memory (1).

Among the signaling cascades the engaged TCR can trigger, the ones able to provide a digital type of signaling, such as Itk/Calcium and ERK (which regulate transcription factors, IRF4 and AP-1 family members) seem to be preferentially involved in promoting terminal effector differentiation (133, 135, 136). Their role in CD8 T_RM_ remains unknown. By contrast, signaling pathways/networks leading to transcription factors that do not strictly fit the rules of TCR signal strength, appear to favor T cell memory fate (BACH2, TCF-1, Eomes) by repressing transcription factors that favor terminal effector differentiation (BACH2 represses AP-1 binding while NR4A1 represses IRF4) (1, 137–146). One of these signals is the NFκB pathway, which appears to be especially critical to the regulation of T cell memory (5, 67, 147). Both, strong and weak TCR signals use this pathway, at least to regulate central memory differentiation (147). NFκB, however, does not seem to regulate the T cell effector versus central memory decision but rather, it controls the survival of CD8 T cells during the transition to memory *via* maintenance of high levels of Eomes and Bcl2, which are crucial for central memory (67, 69, 70). This is possible thanks to a feedback loop where NFκB-Pim1K- Eomes drive a continuum of NFκB signals that extend beyond the peak of the immune response. These proteins also ensure memory maintenance, as memory T cells devoid of either of these failed to survive and respond (67). Whether NFκB signaling has a distinct way to regulate resident memory is unknown. NFκB signaling is also an important driver of inflammation with broad effects. From the induction of pro-inflammatory cytokines (IL-6, etc) to the signaling by inflammatory cytokines (i.e. TNF etc), NFκB holds the potential to inhibit [TGFβ (74)] or potentiate [IL-33 (148)] tissue signals that are essential for CD8 T_RM_ [reviewed in (149)]. Although still unexplored, our previous findings and the fact that Eomes negatively modulates CD8 resident memory development (40), strongly suggest that NFκB may be an important regulator of CD8 T_RM_.

It is also important to mention that TCR signals are not sufficient for CD8 T cell memory and are often tuned by other environmental signals (**Figure 1**). This is the case of inflammatory cytokines IL-12 (102), IL-10 (150) or IL-21 (108) and metabolic signals (151). The metabolic signaling pathway, mTOR, which can also be activated by TCR and IL-12 (152), has been linked to CD8 T_RM_ (153). Although, whether mTOR impacts on migration to tissue and/or T_RM_ survival is still unclear.

Another important question to answer is when antigenic signals are required for establishing resident T memory. Besides the obvious need for antigenic signals to activate naïve T cells, it is widely accepted now that effector T cells that migrate from the draining lymph node to the tissue need to receive a second antigenic hit in the tissue and then, further differentiate into T_RM_ (33, 154). Yet, depending on the tissue the continuous need to maintain antigenic signals to avoid the erosion of T_RM_ remains contentious. Thus, several studies support that antigenic signals are required in brain, lung, female reproductive tract and skin (155–159) to accumulate T_RM_ while in other tissues, re-exposure to antigen may be dispensable (42, 45, 157, 160). These studies only referred to cognate pathogenic antigen and did not address whether local antigenic signals were required once T_RM_ had already been established. Moreover, while it has been shown that CD8 T cell memory does not require self-peptide-MHC signals for its maintenance or establishment (9, 161, 162), the role of self-peptide-MHC in the context of resident memory has not been sufficiently explored yet.

## Conclusion

CD8 T_RM_ are a critical first line of defense against pathogen infections and a promising tool in the fight against tumors. However, the development of CD8 resident memory requires a complex milieu of signals both from the tissues such as TGFβ, IL-33, and IL-15 and from inflammatory cytokines including IL-12 and TNF. Not only are multiple signals required, as this review discusses, specific quantities and timing of the signals are likely to be necessary. While these signals contribute to the development of CD8 resident memory, excessive amounts of some inflammatory cytokines may also limit the differentiation of CD8 T_RM_. Moreover, pharmaceutical treatments such as TNF blockade or other anti-inflammatory regimes may interfere with the development of the regulation of these signals and could possibly alter the development of CD8 T_RM_. As the transcriptional and epigenetic mechanisms that regulate CD8 T_RM_ are becoming clearer, it is also critical that the field puts the effort to fully understand biochemically how tuning antigen, inflammatory and local tissue signals in time affect T_RM_. This information can be extremely valuable to the treatment of diseases where T_RM_ are involved (infection, cancer, autoimmunity, allergies and transplantation).

## Author Contributions

CJP wrote and edited the manuscript as well as organized the review. MAD edited and contributed to the discussion of the manuscript. ET wrote, edited, and contributed to the discussion of the manuscript. All authors contributed to the article and approved the submitted version.

## Funding

This work was supported by grants from the National Institutes of Health (NIH AI110420-01A1, NCI CA244314).

## Conflict of Interest

The authors declare that the research was conducted in the absence of any commercial or financial relationships that could be construed as a potential conflict of interest.
